# Galectin-3 Inhibits Galectin-8/Parkin-Mediated Ubiquitination of Group A Streptococcus

**DOI:** 10.1128/mBio.00899-17

**Published:** 2017-07-25

**Authors:** Yi-Lin Cheng, Yan-Wei Wu, Chih-Feng Kuo, Shiou-Ling Lu, Fu-Tong Liu, Robert Anderson, Chiou-Feng Lin, Yi-Ling Liu, Wan-Yu Wang, Ying-Da Chen, Po-Xing Zheng, Jiunn-Jong Wu, Yee-Shin Lin

**Affiliations:** a Institute of Basic Medical Sciences, College of Medicine, National Cheng Kung University, Tainan, Taiwan; b Department of Microbiology and Immunology, College of Medicine, National Cheng Kung University, Tainan, Taiwan; c Department of Biotechnology and Laboratory Science in Medicine, School of Biomedical Science and Engineering, National Yang-Ming University, Taipei, Taiwan; d Center of Infectious Disease and Signaling Research, National Cheng Kung University, Tainan, Taiwan; e Department of Nursing, I-Shou University, Kaohsiung, Taiwan; f Center for Frontier Oral Science, Graduate School of Dentistry, Osaka University, Osaka, Japan; g Institute of Biomedical Sciences, Academia Sinica, Taipei, Taiwan; h Departments of Microbiology & Immunology and Pediatrics, and Canadian Center for Vaccinology, Dalhousie University, Halifax, Canada; i Department of Microbiology and Immunology, College of Medicine, Taipei Medical University, Taipei, Taiwan; j Graduate Institute of Medical Sciences, College of Medicine, Taipei Medical University, Taipei, Taiwan; University of Maryland, College Park

**Keywords:** galectin-3, galectin-8, group A streptococcus, parkin, ubiquitin

## Abstract

Group A streptococcus (GAS) is an important human pathogen that causes a wide variety of cutaneous and systemic infections. Although originally thought to be an extracellular bacterium, numerous studies have demonstrated that GAS can trigger internalization into nonimmune cells to escape from immune surveillance or antibiotic-mediated killing. Epithelial cells possess a defense mechanism involving autophagy-mediated targeting and killing of GAS within lysosome-fused autophagosomes. In endothelial cells, in contrast, we previously showed that autophagy is not sufficient for GAS killing. In the present study, we showed higher galectin-3 (Gal-3) expression and lower Gal-8 expression in endothelial cells than in epithelial cells. The recruitment of Gal-3 to GAS is higher and the recruitment of Gal-8 to GAS is lower in endothelial cells than in epithelial cells. We further showed that Gal-3 promotes GAS replication and diminishes the recruitment of Gal-8 and ubiquitin, the latter of which is a critical protein for autophagy sequestration. After knockdown of Gal-3 in endothelial cells, the colocalization of Gal-8, parkin, and ubiquitin-decorated GAS is significantly increased, as is the interaction of Gal-8 and parkin, an E3 ligase. Furthermore, inhibition of Gal-8 in epithelial cells attenuates recruitment of parkin; both Gal-8 and parkin contribute to ubiquitin recruitment and GAS elimination. Animal studies confirmed that Gal-3-knockout mice develop less-severe skin damage and that GAS replication can be detected only in the air pouch and not in organs and endothelial cells. These results demonstrate that Gal-3 inhibits ubiquitin recruitment by blocking Gal-8 and parkin recruitment, resulting in GAS replication in endothelial cells.

## IMPORTANCE

In epithelial cells, GAS can be efficiently killed within the lysosome-fused autophaosome compartment. However, we previously showed that, in spite of LC-3 recruitment, the autophagic machinery is not sufficient for GAS killing in endothelial cells. In this report, we provide the first evidence that Gal-3, highly expressed in endothelial cells, blocks the tagging of ubiquitin to GAS by inhibiting recruitment of Gal-8 and parkin, leading to an enhancement of GAS replication. We also provide the first demonstration that Gal-8 can interact with parkin, the critical E3 ligase, for resistance to intracellular bacteria by facilitating the decoration of bacteria with ubiquitin chains. Our findings reveal that differential levels of Gal-3 and Gal-8 expression and recruitment to GAS between epithelial cells and endothelial cells may contribute to the different outcomes of GAS elimination or survival and growth of GAS in these two types of cells.

## INTRODUCTION

Group A streptococcus (GAS), also known as *Streptococcus pyogenes*, causes a wide spectrum of human diseases, ranging from noninvasive infections such as pharyngitis and impetigo to serious systemic infections such as necrotizing fasciitis and streptococcal toxic shock syndrome, as well as autoimmune diseases triggered by repeated infections. In spite of the availability of antibiotics, the incidence of severe invasive GAS infection has continued to increase worldwide in recent years (1–5). Although GAS has been considered to be an extracellular pathogen, the internalization of GAS into nonimmune cells provides a strategy for GAS to evade immune surveillance and antibiotic-mediated killing (6–10).

Autophagy is a membrane trafficking process which leads to the formation of a double-membrane spherical structure called the autophagosome to deliver cytosolic contents into the lysosome followed by their degradation. In addition to the well-understood physiological role of autophagy in recycling cytoplasmic components during nutrient deprivation, increasing evidence reveals that autophagy also plays a role in the innate immune response by targeting intracellular bacteria, a process also known as xenophagy (11–15). Invasive GAS is also targeted by autophagic mechanisms in epithelial cells. After entering epithelial cells, GAS escapes from the endosome into the cytoplasm and is subsequently trapped in autophagosome-like compartments, which in turn fuse with lysosomes, resulting in bacterial killing (15–18). In contrast, our previous study revealed defective autophagosome induction in endothelial cells due to insufficient acidification of the autophagosome, which permits the survival and replication of GAS (19). However, the mechanisms underlying the differences in autophagosome formation between epithelial cells and endothelial cells are not yet clear.

Ubiquitin, a small protein consisting of 76 amino acids, is thought to play an important role in the autophagic sequestration of invading bacteria by targeting pathogens or pathogen-containing vacuoles (20, 21). The adaptor proteins, including p62, nuclear dot protein 52 kDa (NDP52), and optineurin, are further recruited by ubiquitin and microtubule-associated protein 1 light chain 3 (LC3), leading to autophagosome formation (22–25). Although the detailed mechanisms of ubiquitination of bacteria are not fully understood, recent studies have shown that specific E3 ligases, LRSAM1 and parkin, target the surface of bacteria and the membranes of damaged bacterium-containing vesicles, respectively, for ubiquitination (26, 27).

Besides ubiquitin, galectins, a class of beta-galactoside-binding proteins, have also been identified as a tag of invasive bacteria for autophagic machinery targeting. A previous study showed that Gal-8 targeted *Salmonella*-containing damaged vesicles by binding to the glycan exposed on the membrane followed by recruitment of NDP52, which can directly interact with LC3 to promote autophagic defense against intracellular growth of *Salmonella* (28). Another galectin, Gal-3, accumulates at bacterium-containing vesicles, although the role of Gal-3 in autophagy is still unclear. In contrast to Gal-8, Gal-3 provides a survival advantage to the bacterium *Neisseria meningitidis*, whereas bacteremia is decreased in Gal-3-deficient mice (29, 30).

In this study, we investigated the roles of Gal-3 and Gal-8 and their correlation with ubiquitination in endothelial cells and epithelial cells during GAS infection. We found that the levels of Gal-3 protein expression and recruitment to GAS are higher in endothelial cells than in epithelial cells, resulting in a lower level of ubiquitin recruitment and increased bacterial replication by blocking Gal-8, which interacts with the E3 ligase parkin.

## RESULTS

### Endogenous protein expression and recruitment levels of Gal-3 are higher, and those of Gal-8 are lower, in endothelial cells than in epithelial cells.

**FIG 1:**
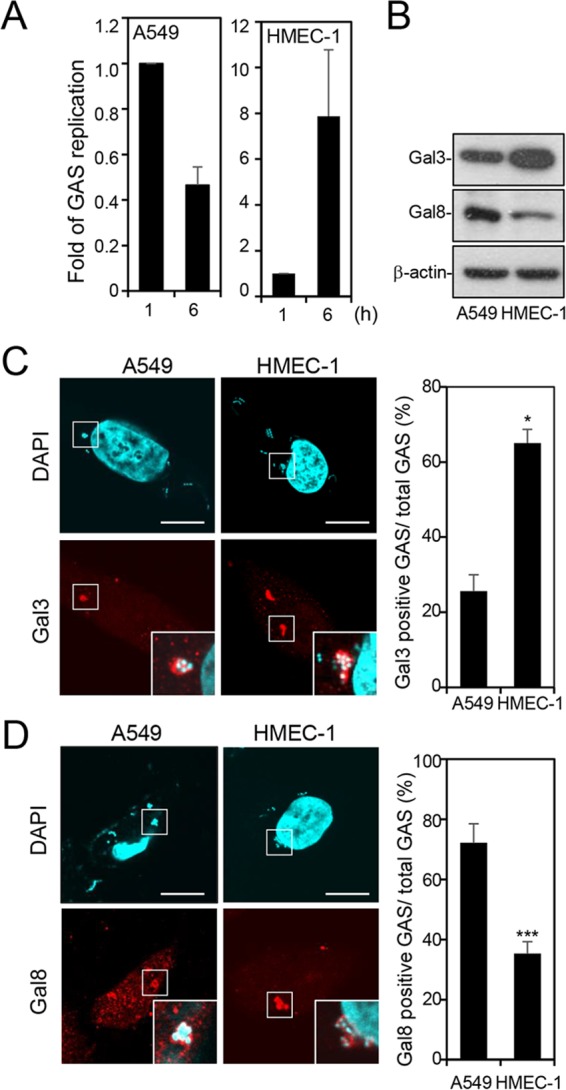
The level of protein expression and recruitment of Gal-3 is higher but that of Gal-8 is lower in HMEC-1 cells than in A549 cells. A549 and HMEC-1 cells were infected with GAS at MOI = 25 and 5, respectively. (A) Cells were infected with GAS for 30 min, and gentamicin was added to kill extracellular bacteria. Cells were collected at 1 and 6 h postinfection. Bacteria were quantified by colony-forming assay, and the fold values of GAS replication were calculated by normalizing the GAS count at 6 h with that at 1 h postinfection. Data represent the means ± SD of results from three independent experiments. (B) Western blot analysis was used to detect the protein expression levels of Gal-3 and Gal-8 in HMEC-1 and A549 cells. (C and D) A549 and HMEC-1 cells were infected with GAS, and gentamicin was added to kill extracellular bacteria. Cells were collected at 1 h postinfection and stained with anti-Gal-3 (C) and anti-Gal-8 (D) antibodies. DAPI was used for cell nuclear and bacterial DNA staining. Bacteria were counted as the number of DAPI-positive particles inside the cells. Images were observed by confocal microscopy. Scale bar, 10 μm (left panel). Levels of GAS surrounded with Gal-3 (C) or Gal-8 (D) were determined relative to total levels of intracellular GAS (right panel). Data represent the means ± SD of results from three independent experiments, and over 100 cells were counted in each sample. *, *P* < 0.05, ***, *P* < 0.001.

To investigate the fate of GAS in epithelial cells and endothelial cells, we infected human lung carcinoma epithelial A549 cells and human microvascular endothelial cell line-1 (HMEC-1) cells with strain NZ131 (M49 serotype) of GAS for 30 min and used gentamicin to kill the extracellular bacteria. By colony-forming assay, we confirmed that GAS replicated in HMEC-1 cells but that its numbers declined in A549 cells (Fig. 1A). Next, we determined the endogenous protein expression of Gal-3 and Gal-8 in A549 cells and HMEC-1 cells. Results showed that HMEC-1 cells contained a higher level of Gal-3 and a lower level of Gal-8 than A549 cells (Fig. 1B). Both Gal-3 and Gal-8 expression levels did not change after GAS infection at various time periods in either HMEC-1 cells or A549 cells (see Fig. S1 in the supplemental material). To further determine the relative levels of recruitment of Gal-3 and Gal-8 to GAS, cells infected with GAS for 1 h were immunostained and observed by confocal microscopy. The imaging results showed that Gal-3 and Gal-8 were recruited to GAS in both cell types (Fig. 1C and D) but that the percentage of Gal-3-positive GAS in HMEC-1 cells was higher than in A549 cells (Fig. 1C) and the percentage of Gal-8-positive GAS in HMEC-1 cells was lower than in A549 cells (Fig. 1D). We also performed double staining of Gal-3 and Gal-8 followed by confocal analysis in A549 and HMEC-1 cells. The results confirmed that Gal-3-positive GAS predominated in HMEC-1 cells, whereas Gal-8-positive GAS predominated in A549 cells (Fig. S2). These results revealed that the levels of protein expression and recruitment of Gal-3 are higher, but those of Gal-8 are lower, in endothelial cells than in epithelial cells.

10.1128/mBio.00899-17.1 FIG S1  GAS infection does not change the endogenous expression of Gal-3 and Gal-8. A549 and HMEC-1 cells were infected with GAS at MOI = 25 and 5, respectively. Cells were infected with GAS for 30 min, and gentamicin was added to kill extracellular bacteria. Cells were collected at indicated time points. Western blot analysis was used to detect the protein expression levels of Gal-3 and Gal-8 in HMEC-1 and A549 cells. Download FIG S1, TIF file, 0.3 MB. Copyright © 2017 Cheng et al. 2017 Cheng et al. This content is distributed under the terms of the Creative Commons Attribution 4.0 International license.

10.1128/mBio.00899-17.2 FIG S2  Predominance of Gal-8-decorated GAS in A549 cells but of Gal-3-decorated GAS in HMEC-1 cells. A549 and HMEC-1 cells were infected with GAS at MOI = 25 and 5, respectively. (A to D) A549 cells (A and B) and HMEC-1 cells (C and D) were infected with GAS, and gentamicin was added to kill extracellular bacteria. Cells were collected at 1 h postinfection and stained with anti-Gal-3 and anti-Gal-8 antibodies. DAPI was used for cell nuclear and bacterial DNA staining. Bacteria were counted as DAPI-positive particles inside the cells. Images were observed by confocal microscopy. Scale bar, 10 μm. (E) Levels of GAS surrounded with Gal-3 or Gal-8 were determined relative to total levels of intracellular GAS. All quantitative data represent the means ± SD of results from three independent experiments, and over 100 cells were counted in each sample. ***, *P* < 0.001. Download FIG S2, TIF file, 4.1 MB. Copyright © 2017 Cheng et al. 2017 Cheng et al. This content is distributed under the terms of the Creative Commons Attribution 4.0 International license.

### Gal-3 enhances GAS replication in endothelial cells.

**FIG 2:**
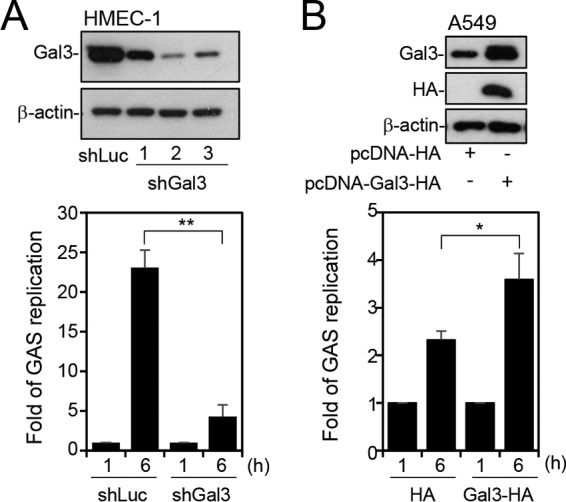
Gal-3 promotes GAS replication. (A) Gal-3 expression was silenced in HMEC-1 cells by using three lentivirus-based shRNAs (shGal3 clones 1, 2, and 3). Luciferase shRNA (shLuc) was used as a negative control. The expression of Gal-3 was detected by Western blot analysis (upper panel). shLuc and shGal3 (clone 2) cells were further infected with GAS at MOI = 5 for 30 min, and gentamicin was added to kill extracellular bacteria. Cells were collected at 1 and 6 h postinfection. The colony-forming assay was performed, and the fold values of GAS replication were calculated by normalizing the GAS count at 6 h with that at 1 h postinfection (lower panel). Data represent the means ± SD of results from three independent experiments. **, *P* < 0.01. (B) A549 cells were transfected with pcDNA-HA or pcDNA-Gal3-HA. Western blot analysis was used to detect the expression of Gal-3 and HA (upper panel). The transfected cells were further infected with GAS at MOI = 25 for 30 min, and gentamicin was added to kill extracellular bacteria. Cells were collected at 1 and 6 h postinfection. Bacteria were counted by colony-forming assay, and the fold values of GAS replication were calculated by normalizing the GAS count at 6 h with that at 1 h postinfection (lower panel). Data represent the means ± SD of results from three independent experiments. *, *P* < 0.05.

To further explore the role of Gal-3 in GAS replication, we used lentiviral short hairpin RNA (shRNA) to establish Gal-3-deficient HMEC-1 cells (shGal3-HMEC-1 cells). By Western blot analysis, clone 2 of shGal3-HMEC-1 cells showed the highest knockdown efficiency (Fig. 2A, upper panel). We therefore infected shGal3-HMEC-1 cells (clone 2) and control HMEC-1 cells transduced with luciferase shRNA (shLuc-HMEC-1 cells) with GAS and examined the replication of GAS inside the cells by colony-forming assay. The results showed that GAS replication was significantly reduced in shGal3-HMEC-1 cells compared with shLuc-HMEC-1 cells (Fig. 2A, lower panel). Overexpression of Gal-3 by transfection with plasmid pcDNA-Gal3-HA, which was confirmed by determining the expression of Gal-3 and hemagglutinin (HA) (Fig. 2B, upper panel), promoted the replication of GAS in A549 cells (Fig. 2B, lower panel). GAS replication was also enhanced by transfection with pEGFP-Gal3 compared with pEGFP-N1 in A549 cells (see Fig. S3 in the supplemental material). In contrast, transfection of A549 cells with control plasmids pcDNA-HA (Fig. 2B, lower panel) and pEGFP-N1 (see Fig. S3B in the supplemental material) resulted in only a slight increase in bacterial numbers at 6 h. These data indicate that Gal-3 has the ability to promote GAS replication.

10.1128/mBio.00899-17.3 FIG S3  Overexpression of Gal-3 promotes GAS replication. A549 cells were transfected with pEGFP-N1 or pEGFP-Gal3. Western blot analysis was used to detect the expression of EGFP-Gal-3 and EGFP (left panel). The transfected cells were further infected with GAS at MOI = 25 for 30 min, and gentamicin was added to kill extracellular bacteria. Cells were collected at 1 and 6 h postinfection. Bacteria were quantified by colony-forming assay, and the fold values of GAS replication were calculated by normalizing the GAS count at 6 h with that at 1 h postinfection (lower panel). Data represent the means ± SD of results from three independent experiments. *, *P* < 0.05. Download FIG S3, TIF file, 0.2 MB. Copyright © 2017 Cheng et al. 2017 Cheng et al. This content is distributed under the terms of the Creative Commons Attribution 4.0 International license.

### Gal-3 blocks the recruitment of ubiquitin and Gal-8 to GAS.

**FIG 3:**
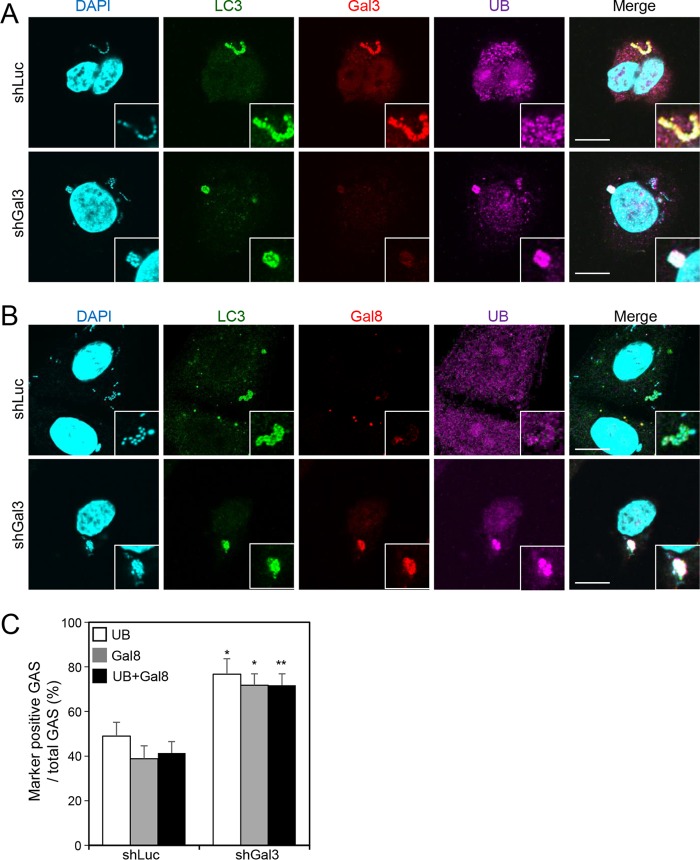
Knockdown of Gal-3 increases recruitment of ubiquitin and Gal-8 to GAS. (A and B) shLuc-HMEC-1 and shGal3-HMEC-1 cells were infected with GAS at MOI = 5 for 30 min, and gentamicin was added to kill extracellular bacteria. Cells were collected at 1 h postinfection and stained with anti-LC3, anti-Gal-3 (A), anti-Gal-8 (B), and anti-ubiquitin (UB) antibodies. DAPI was used for cell nuclear and bacterial DNA staining. Images were observed by confocal microscopy. Scale bar, 10 μm. (C) Levels of GAS surrounded with UB and Gal-8 were determined relative to total levels of intracellular GAS. All quantitative data represent the means ± SD of results from three independent experiments, and over 100 cells were counted in each sample. *, *P* < 0.05, **, *P* < 0.01 (compared to shLuc cells).

Ubiquitination is critical for clearance of invading bacteria by recruiting autophagic machinery (20, 21). In order to clarify whether the enhancement of GAS replication by Gal-3 occurred by blocking ubiquitination, we monitored the recruitment of ubiquitin by confocal microscopy after knockdown of Gal-3 in HMEC-1 cells. In the representative image of the shLuc-HMEC-1 cell, in spite of LC3 recruitment, ubiquitin was not recruited to Gal-3-decorated GAS (Fig. 3A, upper panel). However, ubiquitin was efficiently recruited to GAS in the low-Gal-3-expressing shGal-3 cells (Fig. 3A, lower panel). Furthermore, not only ubiquitin but also Gal-8 was recruited to GAS after depletion of Gal-3 (Fig. 3B, lower panel). The quantitative results showed that almost all the detectable Gal-8 coexpressed with ubiquitin-positive GAS and that the overall level was increased in Gal-3-depleted cells (Fig. 3C). These results demonstrate that Gal-3 attenuates the recruitment of Gal-8 and ubiquitin to GAS.

### Gal-3 inhibits ubiquitin recruitment by blocking parkin, which directly interacts with Gal-8.

**FIG 4:**
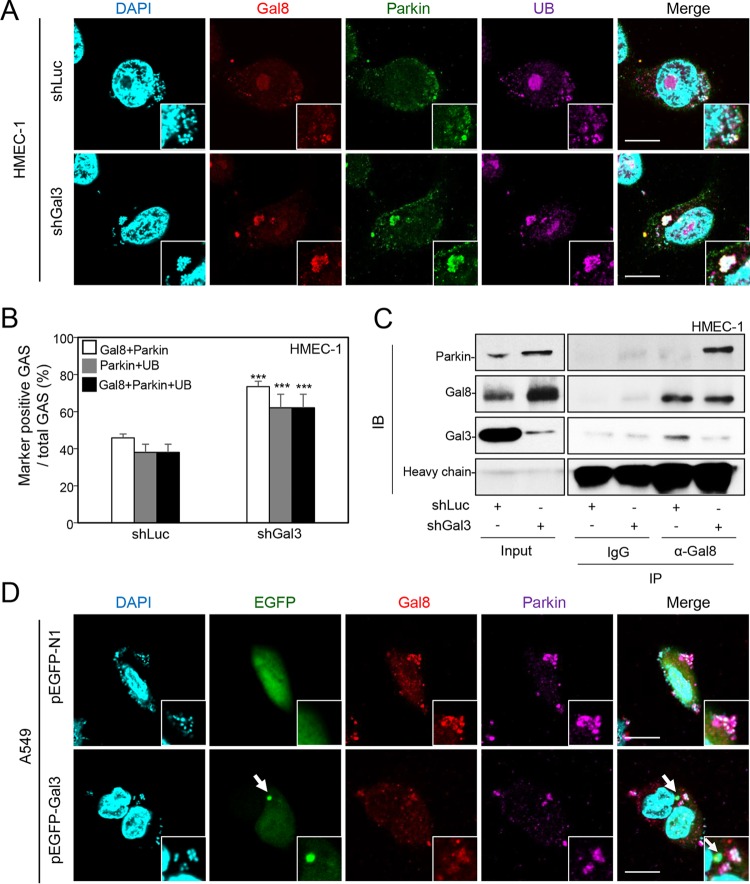
Gal-3 inhibits recruitment of Gal-8 and parkin, which interact with each other. (A) shLuc-HMEC-1 and shGal3-HMEC-1 cells were infected with GAS at MOI = 5 for 30 min, and gentamicin was added to kill extracellular bacteria. Cells were collected at 1 h postinfection and stained with anti-Gal-8, anti-parkin, and anti-ubiquitin (UB) antibodies. DAPI was used for cell nuclear and bacterial DNA staining. Images were observed by confocal microscopy. Scale bar, 10 μm. (B) Levels of GAS surrounded with Gal-8, parkin, and UB were determined relative to total levels of intracellular GAS. All quantitative data represent the means ± SD of results from three independent experiments, and over 100 cells were counted in each sample. ***, *P* < 0.001 (compared to shLuc cells). (C) shLuc-HMEC-1 and shGal3-HMEC-1 cells were infected with GAS at MOI = 5 for 30 min, and gentamicin was added to kill extracellular bacteria. Cells were collected at 1 h postinfection, and immunoprecipitation (IP) was performed using anti-Gal-8 antibody and goat control IgG followed by detection of parkin, Gal-8, and Gal-3 by Western blotting. The loading control was either whole-cell stain (Input) or IgG heavy chain. IB, immunoblot. (D) A549 cells were transfected with pEGFP-N1 or pEGFP-Gal3. Cells were further infected with GAS at MOI = 25 for 30 min, and gentamicin was added to kill extracellular bacteria. Cells were collected at 1 h postinfection and stained with anti-Gal-8 and anti-parkin antibodies. DAPI was used for cell nuclear and bacterial DNA staining. Images were observed by confocal microscopy. Scale bar, 10 μm.

A previous study showed that parkin, an E3 ligase, is responsible for catalyzing the polyubiquitin chain surrounding *Mycobacterium tuberculosis*, resulting in inhibition of bacterial growth (27). We therefore hypothesized that Gal-8, which is blocked by Gal-3, may interact with parkin to recruit ubiquitin. To test this hypothesis, we determined the expression of parkin around GAS after depletion of Gal-3. The results of confocal microscopy revealed that Gal-8, parkin, and ubiquitin were recruited to GAS efficiently in Gal-3-deficient endothelial cells (Fig. 4A, lower panel). Levels of Gal-8 and parkin double-positive GAS, parkin and ubiquitin double-positive GAS, and Gal-8, parkin, and ubiquitin triple-positive GAS all increased in Gal-3-knockdown HMEC-1 cells (Fig. 4B). To further investigate whether Gal-8 interacted with parkin directly and whether the interaction was regulated by Gal-3, the lysates of shLuc-HMEC-1 and shGal3-HMEC-1 cells infected with GAS were immunoprecipitated with anti-Gal-8 antibody and then analyzed for interaction with parkin by Western blotting. The results showed that knockdown of Gal-3 markedly enhanced the interaction of parkin with Gal-8 (Fig. 4C). The interaction of Gal-3 and Gal-8 could also be observed and was markedly reduced in Gal-3-knockdown cells compared with shLuc control cells (Fig. 4C). The results suggest that Gal-8, if not blocked by Gal-3, recruits and binds to parkin directly. Furthermore, in the Gal-3-transfected A549 cells, the Gal-3-decorated GAS (as indicated by concentrated green fluorescence of enhanced green fluorescent protein [EGFP]) did not express Gal-8 or parkin (Fig. 4D, lower panel, arrow). The recruitment of parkin and ubiquitin was also blocked by overexpressing EGFP-Gal-3 (see Fig. S4 in the supplemental material).

10.1128/mBio.00899-17.4 FIG S4  Overexpression of Gal-3 blocks the recruitment of parkin and ubiquitin. A549 cells were transfected with pEGFP-N1 or pEGFP-Gal3. Cells were further infected with GAS at MOI = 25 for 30 min, and gentamicin was added to kill extracellular bacteria. Cells were collected at 1 h postinfection and stained with anti-parkin and anti-ubiquitin (UB) antibodies. DAPI was used for cell nuclear and bacterial DNA staining. Images were observed by confocal microscopy. Scale bar, 10 μm. Download FIG S4, TIF file, 1.6 MB. Copyright © 2017 Cheng et al. 2017 Cheng et al. This content is distributed under the terms of the Creative Commons Attribution 4.0 International license.

### Gal-8 and parkin contribute to ubiquitin recruitment and repress GAS replication, whereas Gal-8 deficiency downregulates recruitment of parkin.

**FIG 5:**
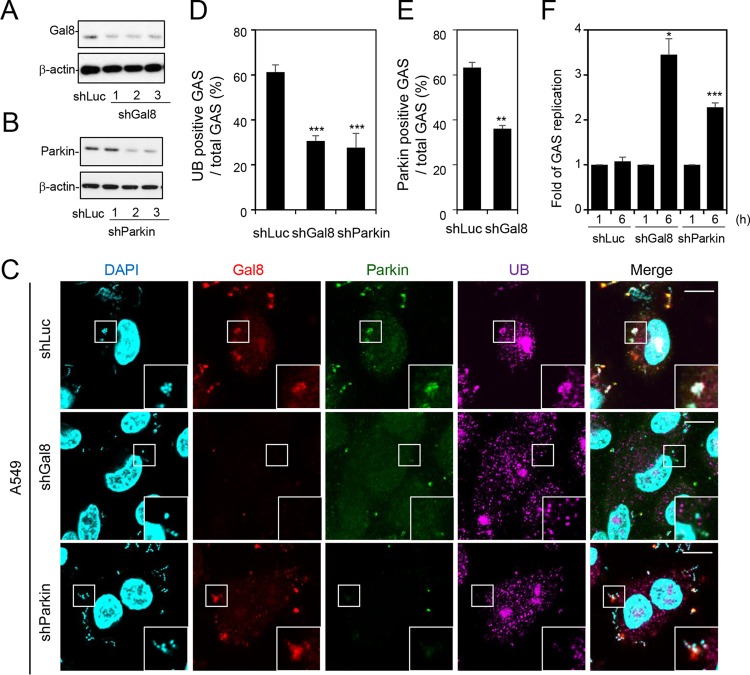
Both Gal-8 and parkin contribute to ubiquitin recruitment and GAS elimination, and Gal-8 knockdown reduces recruitment of parkin to GAS. (A and B) Gal-8 (A) and parkin (B) expression was silenced in A549 cells using three lentivirus-based shRNAs (shGal8 clones 1, 2, and 3 and shParkin clones 1, 2, and 3). Luciferase shRNA (shLuc) was used as a negative control. The expression of Gal-8 and parkin was detected by Western blot analysis. (C) shLuc-A549, shGal8-A549 (clone 2), and shParkin-A549 (clone 2) cells were infected with GAS at MOI = 25 for 30 min, and gentamicin was added to kill extracellular bacteria. Cells were collected at 1 h postinfection and stained with anti-Gal-8, anti-parkin, and anti-ubiquitin (UB) antibodies. DAPI was used for cell nuclear and bacterial DNA staining. Images were observed by confocal microscopy. Scale bar, 10 μm. (D and E) Levels of GAS surrounded with UB (D) and parkin (E) were determined relative to total levels of intracellular GAS. All quantitative data represent the means ± SD of results from three independent experiments, and over 100 cells were counted in each sample. **, *P* < 0.01, ***, *P* < 0.001 (compared to shLuc cells). (F) shLuc-A549, shGal8-A549, and shParkin-A549 cells were further infected with GAS at MOI = 25 for 30 min, and gentamicin was added to kill extracellular bacteria. Cells were collected at 1 and 6 h postinfection. Bacteria were quantified by colony-forming assay, and the fold values of GAS replication were calculated by normalizing the GAS count at 6 h with that at 1 h postinfection. Data represent the means ± SD of results from three independent experiments. *, *P* < 0.05, ***, *P* < 0.001 (compared to shLuc cells).

Since Gal-8 colocalized with parkin and ubiquitin, we next confirmed the role of Gal-8 and parkin in ubiquitin recruitment and GAS replication. We first reduced expression of Gal-8 and parkin in A549 cells by lentivirus-based shRNA knockdown and checked the knockdown efficiency by Western blot analysis. On the basis of the Western blotting results, we chose clone 1 of shGal-8 cells and clone 2 of shParkin cells to perform the experiments whose results are shown in Fig. 5A and B. After downregulation of Gal-8 or parkin in A549 cells, the recruitment of ubiquitin was significantly reduced (Fig. 5C and D). In addition, knockdown of Gal-8 also decreased the level of parkin-positive GAS in A549 cells (Fig. 5C and E). Concomitant with the inhibition of ubiquitin recruitment, the replication of GAS was increased in Gal-8- and parkin-deficient cells (Fig. 5F). Therefore, we conclude that Gal-8 and parkin play important roles in ubiquitin recruitment as well as in GAS elimination in epithelial cells.

### Gal-3-knockout mice develop less-severe skin damage and show lower levels of bacterial replication than wild-type mice.

**FIG 6:**
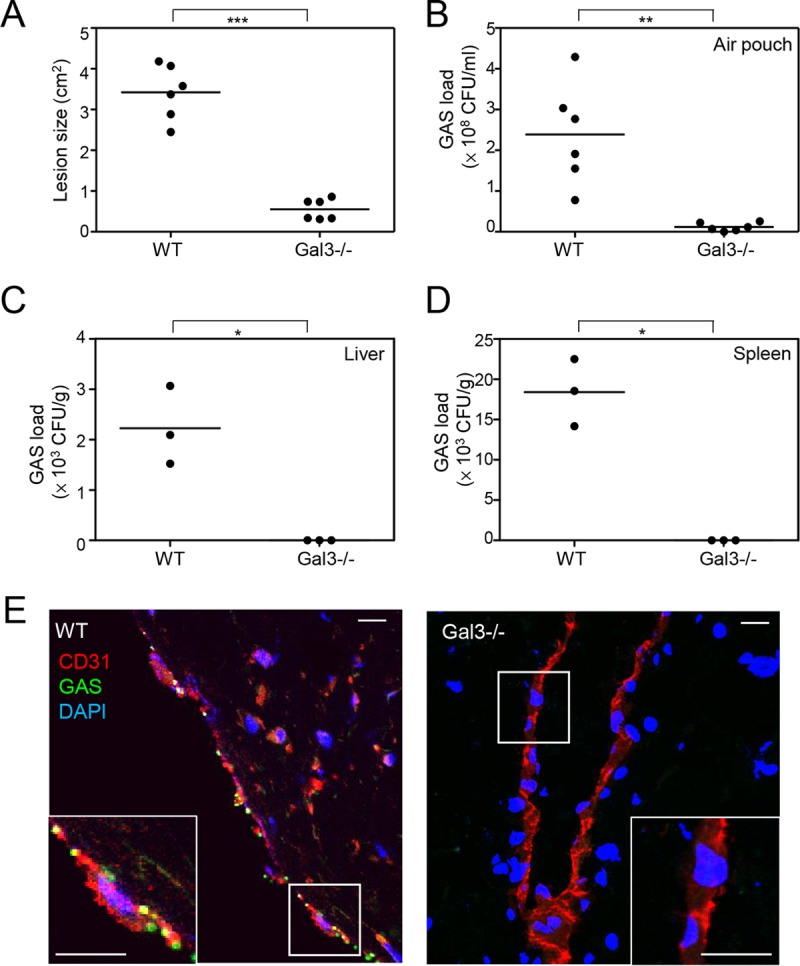
GAS-induced skin damage and GAS replication are reduced in Gal3^−/−^ mice compared to wild-type mice. C57BL/6JNarl wild-type (WT) and Gal-3-knockout (Gal-3^−/−^) mice were inoculated with GAS in the air pouch. All samples were collected at 48 h postinfection. (A) The diapedetic areas were photographed and measured by ImageJ. (B to D) Bacterial counts of air pouch exudates (B), liver (C), and spleen (D) were determined by plating, and the data were pooled from three independent experiments. *, *P* < 0.05; **, *P* < 0.01; ***, *P* < 0.001. (E) The cryosectioned skin tissues were stained with anti-GAS and anti-CD31 antibodies. DAPI was used for cell nuclear staining. Images were observed by confocal microscopy. Scale bar, 10 μm.

To further illustrate the role of Gal-3 in GAS infection *in vivo*, we challenged Gal-3-knockout (Gal-3^−/−^) mice and wild-type (WT) mice with GAS through air pouch inoculation. At 48 h postinfection, we measured skin lesions by the use of ImageJ. As shown in the representative results, the diapedetic area of WT mice was larger than that of Gal-3^−/−^ mice (see Fig. S5 in the supplemental material). The average lesion was smaller in Gal3^−/−^ mice than in WT mice (Fig. 6A). The bacterial counts in the air pouch exudate and the homogenate of liver and spleen were further determined by plating. The results revealed that growth of GAS in the air pouch was reduced in Gal-3^−/−^ mice compared with WT mice (Fig. 6B). Furthermore, GAS was detected in the liver and spleen of WT mice but not Gal-3^−/−^ mice (Fig. 6C and D). We further stained GAS and endothelial cells with anti-GAS antibody and anti-CD31 antibody, respectively, in skin cryosections. The results showed that GAS could be detected in the endothelial cells of WT mice but not Gal-3^−/−^ mice (Fig. 6E). Taken together, the *in vivo* results suggest that Gal-3 promotes GAS replication in endothelial cells, leading to bacterial dissemination to the organs.

10.1128/mBio.00899-17.5 FIG S5  Gal3^−/−^ mice develop less-severe skin damage than wild-type mice. C57BL/6JNarl wild-type (WT) and Gal-3-knockout (Gal-3^−/−^) mice were inoculated with GAS in the air pouch. The diapedetic areas were photographed and measured by ImageJ at 48 h postinfection. Download FIG S5, TIF file, 0.9 MB. Copyright © 2017 Cheng et al. 2017 Cheng et al. This content is distributed under the terms of the Creative Commons Attribution 4.0 International license.

## DISCUSSION

**FIG 7:**
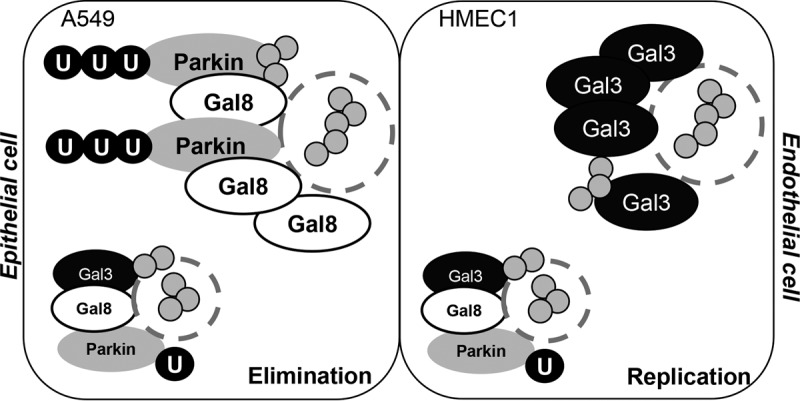
A hypothetical model of the regulation of Gal-3 and Gal-8 in GAS replication in epithelial cells and endothelial cells. Higher levels of expression and recruitment of Gal-3 in endothelial cells inhibit the recruitment of Gal-8 and parkin to GAS, resulting in less ubiquitin recruitment and more GAS replication. In contrast, higher levels of expression and recruitment of Gal-8 in epithelial cells inhibit the recruitment of Gal-3 but induce the recruitment of parkin, resulting in higher ubiquitin recruitment and GAS elimination.

In the present study, we showed that Gal-3 is a critical factor for GAS replication in endothelial cells. Higher levels of expression and recruitment of Gal-3 to GAS in endothelial cells than in epithelial cells may compete for the binding of Gal-8 and the recruitment of parkin, resulting in inhibition of ubiquitination and enhancement of bacterial replication. In epithelial cells, however, ubiquitin efficiently localizes to GAS owing to higher expression and recruitment of Gal-8, which further interacts with parkin and promotes GAS elimination (Fig. 7). Furthermore, the animal studies showed a lack of bacteria in the liver, spleen, and endothelial cells of Gal-3^−/−^ mice, suggesting that deficiency of Gal-3 may inhibit GAS replication and its subsequent dissemination systemically. Although there is still no direct clinical evidence for differential levels of susceptibility of endothelial cells and epithelial cells to GAS, the breaking down of blood vessel barriers, which are composed of endothelial cells, is a key step in the dissemination of GAS from tissue to the blood circulation. Furthermore, several clinical strains of GAS which are commonly associated with invasive GAS infection have been shown to internalize into primary endothelial cells *in vitro* (31, 32).

All galectins show modulation of their expression during development, differentiation, and some physiological and pathological states (33). Some studies have reported that transcriptional regulation and DNA methylation can participate in the regulation of galectins (33, 34). For example, the genomic region of human Gal-3, which has promoter activity, has been characterized, and several putative transcription factor binding sites, including the binding sites of nuclear factor (NF)-κB, activator protein-1 (AP-1), and cyclic AMP (cAMP) response element binding protein (CREB), have been identified in this promoter region (35). Although more investigation is required, factors such as transcriptional regulation or DNA methylation may vary in endothelial and epithelial cell lineages, resulting in different expression levels of Gal-3 and Gal-8.

Ubiquitination is well recognized as an initial step for specific targeting of selective autophagy, including xenophagy and mitophagy (21, 36). In GAS infection of epithelial cells, during which cytoplasmic bacteria are efficiently delivered to autophagolysosomes for degradation, NDP52 has been shown to be important in bacterial clearance via its linkage between ubiquitin and LC3 (24). However, the role of ubiquitin in GAS eradication is still not clear. Ubiquitination is a complex process which involves more than a hundred combinations of E1 activating enzymes, E2 conjugate enzymes, and E3 ligases and generates over 10 types of ubiquitin chains on various targets (37). Parkin, an E3 ligase, with a well-established role in mitophagy, mediates resistance to intracellular growth of *M. tuberculosis* in macrophages (27). In the present study, we show that parkin is involved in GAS ubiquitination and is required for GAS suppression in both epithelial cells and endothelial cells. However, under conditions of high Gal-3 expression levels, the recruitment of parkin by Gal-8 was lower in endothelial cells than in epithelial cells.

Although the detailed mechanisms of the regulation seen between Gal-3 and Gal-8 remain to be determined, the results presented in Fig. S2 in the supplemental material and Fig. 4C suggest the possibility of effects of competition between Gal-3 and Gal-8. In A549 cells, more than 40% of GAS were surrounded by Gal-8 alone and about 20% of GAS were doubly positive for Gal-3 and Gal-8. Notably, very limited amounts of GAS were surrounded by Gal-3 alone. However, in HMEC-1 cells, 30% of GAS were surrounded by Gal-3 alone, and about 30% of GAS were doubly positive for Gal-3 and Gal-8. Only very few GAS were surrounded by Gal-8 alone (Fig. S2E). These results suggest that high levels of Gal-8 binding (with or without Gal-3) and low levels of binding by Gal-3 alone (without Gal-8) to GAS in A549 cells result in inhibition of GAS replication. In HMEC-1 cells, however, the level of GAS surrounded by Gal-3 alone (i.e., without Gal-8) was sufficient for GAS replication.

Galectins are synthesized as cytosolic proteins, but they can also be secreted from cells via a nonclassical pathway. Through binding to glycans, galectins modulate multiple cellular functions (38–40). In bacterial infection, recent reports showed that galectins can sense the exposure of host glycans on ruptured membranes of bacterium-containing vesicles. Gal-3, Gal-8, and Gal-9 are recruited to *Salmonella*-, *Shigella*-, or *Listeria*-containing vesicles in epithelial cells, while Gal-1, Gal-3, and Gal-8 may also accumulate in damaged lysosomes (28, 41, 42). In addition to cellular glycans, the bacterial surface is extensively covered by a polysaccharide capsule, which may provide common structures similar to those seen with certain host glycans. Therefore, the biological functions of galectins may also be mediated through recognition of bacterial surface glycans (42, 43). It has been reported that Gal-3, Gal-4, and Gal-8 can bind to the *Escherichia coli* O86 surface glycan, resulting in bacterial killing (28, 44). Recognition of Gal-3 on the bacterial surface also inhibits the replication of *Streptococcus pneumoniae* (45). In other situations, the interaction between galectins and bacteria can be beneficial to the bacteria. For example, Gal-3 enhances the attachment of *Helicobacter pylori* to host cells (46). Binding of Gal-3 diminished the recognition of *M. tuberculosis* by dendritic cells and macrophages through Fc receptors (47). GAS also possesses a polysaccharide capsule expressing strain-dependent variations in carbohydrate chains. The glycan-binding specificities of Gal-3 and Gal-8 in GAS recruitment require further investigation. While parkin is responsible for catalyzing Lys63-linked polyubiquitination of bacterium-containing phagosome membrane segments (27), our immunoprecipitation results demonstrated that Gal-8 interacts directly with parkin. In addition to binding to bacteria, galectins may also sense host glycans on the membrane of GAS-containing damaged vesicles.

Galectins specifically recognize β-galactoside-containing glycans which are expressed in extracellular compartments but also recognize the lumen of intracellular vesicles, such as endosomes (48). Although all galectins can recognize some basic glycan structures, such as Galβ1-4GlcNAc, or a linkage isomer, Galβ1-3GlcNAc, the affinity for these glycans differs among the galectins (49, 50). For example, Gal-3 contains the highest affinity for these two basic structures. Moreover, galectins also show different sugar-binding affinities and specificities for “branched,” “repeated,” and “substituted” glycans (50, 51). Although the glycan composition of the GAS-containing endosome is not fully understood, the galectin-binding glycans which originally existed in extracellular compartments may be internalized into the endosome and influence the competition between Gal-3 and Gal-8 on the damaged endosome membrane. Furthermore, previous reports showed that Gal-3 and Gal-8 can bind to the same extracellular matrix proteins, such as integrin αM (52, 53). Gal-3 can even form pentamers upon binding to multivalent targets and occupies considerable space by its three-dimensional structure (54). We hypothesize that there is a competitive effect of highly expressed Gal-3 on Gal-8, but the detailed mechanisms remain to be determined.

In addition to regulation of Gal-8/parkin, other Gal-3-related machinery involved in GAS defense may also exist. The common role of Gal-3 in promoting infection by bacteria such as *Neisseria meningitidis* and *Pseudomonase aeruginosa* is to enhance pathogen attachment to or entry into the host cells (30, 55). A recent report revealed that intrinsic Gal-3 mediates resistance of *Candida* to neutrophil reactive oxygen species (ROS)-dependent killing by modulating complement receptor 3 downstream syk kinase activation (56). ROS, which is also produced by immune cells during GAS infection, can damage bacterial nucleic acids, cell membranes, and proteins. Because GAS may encounter ROS in multiple stages of infection, GAS has developed an array of proteins and virulence factors for defense against oxidative stress (57). Based on those studies, Gal-3 may also enhance GAS attachment to host cells extracellularly and may help GAS survive ROS intracellularly.

Several kinds of bacteria have been reported to induce expression of Gal-3, including *H. pylori*, *Streptococcus pneumoniae*, *Francisella*, and *Corynebacterium kutscheri* (46, 58–60). Among them, *H. pylori* has been demonstrated to induce upregulation and secretion of Gal-3 through the mitogen-activated protein kinase (MAPK)-mediated pathway. Gal-3 binds to O-antigen of *H. pylori* and promotes adhesion of bacteria to the cell surface (46). Although we did not observe induction of Gal-3 after 6 h of GAS infection *in vitro* (Fig. S1), there are still other factors which may be involved in the induction of Gal-3 after GAS infection *in vivo*. A previous study revealed that Gal-3 is upregulated by NF-κB or hypoxia-inducible factor-1-alpha (HIF-1α) under several conditions, such as hypoxia or nutrient deprivation (61). These two transcription factors are also activated by GAS infection (62–65). However, whether NF-κB- or HIF-1α-mediated Gal-3 upregulation is involved in GAS infection and what the inducer of these two transcription factors is still need further investigation.

A recent report showed that Gal-8 targeted *Salmonella*-containing damaged vesicles followed by recruitment of NDP52, which can directly interact with LC3 to promote autophagic defense against *Salmonella* (28). Although Gal-8 can recruit LC3 through interaction with NDP52, the interaction of Gal-8 and the details of ubiquitination have not been reported. Here, we provide the first evidence that Gal-8 can recruit and interact with E3 ligase parkin. It has also been reported that NDP52 directly interacts with E3 ligase LRSAM1 but is not required for recruitment of LRSAM1 (26). Apart from parkin, the recently elucidated proteome-scale map of the human interactome shows that Gal-8 can directly interact with an E3 ligase, tripartite motif protein 23 (TRIM23) (66). Although the role of TRIM23 in autophagy is still largely unknown, TRIM family proteins have been reported to act as autophagy receptors or as the platform for assembly of the core autophagy regulators such as unc-51-like autophagy activating kinase 1 (ULK1) and Beclin 1 in their activated state (67). Whether TRIM23 or other E3 ligases can interact with Gal-8 and play a role in promoting autophagic GAS clearance is an important issue for future studies.

## MATERIALS AND METHODS

### Cell culture.

Cells of human microvascular endothelial cell line-1 (HMEC-1), obtained from the Centers for Disease Control and Prevention, USA, were grown in culture plates containing endothelial cell growth medium M200 (Cascade Biologics) composed of 10% fetal bovine serum (FBS), 1 μg/ml hydrocortisone, 10 ng/ml epidermal growth factor, 3 ng/ml basic fibroblast growth factor, and 10 μg/ml heparin. HMEC-1 cells retain the morphological, phenotypic, and functional characteristics of normal human microvascular endothelial cells (68). Human lung carcinoma epithelial A549 cells were maintained in Dulbecco’s modified Eagle’s medium (DMEM) supplemented with 10% FBS. Cells were cultured at 37°C in 5% CO_2_ and detached with 1,000 U/ml trypsin and 0.5 mM EDTA for passage. Once cell confluence reached 80%, the cells were detached with trypsin-EDTA and seeded at 8 × 10^4^ cells in 24-well plates for colony-forming assay and at 6 × 10^4^ cells in 24-well plates with cover glass for fluorescence microscope observation.

### Bacteria.

GAS strain NZ131 (M49 serotype) was a gift from D. R. Martin (New Zealand Communicable Disease Centre, Porirua) (69). GAS grew at 37°C in tryptic soy broth containing 0.5% yeast extract (TSBY) overnight and was transferred to fresh broth at a 1:50 dilution for 3 h (refreshed GAS). The refreshed, early log GAS was used as the standard culture for the experiments. The bacteria were harvested by centrifugation (3,500 rpm, 10 min, 4°C) and resuspended in phosphate-buffered saline (PBS), and the concentration was determined by spectrophotometry using an optical density at 600 nm (OD_600_) of 0.2 as 1 × 10^8^ CFU/ml and confirmed by viable-colony counting.

### Infection model.

Cells at 80% confluence were plated in 24-well plates or 6-well plates and incubated overnight. The prepared bacteria were directly added into wells at various multiplicities of infection (MOI). In order to ensure simultaneous infection of cells, the plates were centrifuged at 500 × *g* for 5 min at 4°C. After a 30-min incubation, the cell culture was washed three times with PBS, and fresh medium containing 100 μg/ml gentamicin was added to kill extracellular bacteria. The cells were collected after various periods of time as indicated in each experiment.

### Immunofluorescence staining.

Cells were seeded at 6 × 10^4^ in 24-well plates with cover glass for overnight culture and infected with GAS for 30 min. Extracellular bacteria were killed by the use of 100 μg/ml gentamicin. At various time points postinfection, the cells were fixed with 4% paraformaldehyde, permeabilized with 0.1% Triton X-100, and stained with anti-LC3 (pM036; MBL), anti-Gal-3 (M3/38; Santa Cruz), anti-Gal-8 (D-18; Santa Cruz), anti-ubiquitin (ab7780; Abcam, Inc.), and anti-parkin (PRK8; Santa Cruz) antibodies at room temperature for 1 h. After the cells were washed with PBS, they were stained with Alexa Fluor-conjugated secondary antibodies and DAPI (4′,6-diamidino-2-phenylindole) for 1 h, and the samples were then analyzed by confocal microscopy (FV1000; Olympus).

### Colony-forming assay.

Cells were seeded at 1 × 10^5^/well in 24-well plates overnight and infected with GAS. Extracellular bacteria were killed by the use of gentamicin. After various time periods, cells were washed twice with PBS and resuspended in 1-ml sterile water for 10 min. The cell lysates were plated using serial dilution on TSBY agar plates. Colonies were grown and counted after 24 h of culture.

### Western blotting.

Harvested cells were lysed in a buffer containing 1% Triton X-100, 50 mM Tris (pH 7.5), 10 mM EDTA, 0.02% NaN_3_, and a protease inhibitor cocktail (Roche Boehringer Mannheim Diagnostics, Mannheim, Germany). After a freeze-thaw cycle, cell lysates were centrifuged at 10,000 × *g* and 4°C for 20 min. The lysates were boiled in sample buffer for 5 min. Samples were then subjected to SDS-PAGE and proteins transferred to polyvinylidene difluoride (PVDF) membranes (Millipore) using a semidry electroblotting system. After blocking with 5% skim milk–PBS was performed, the membranes were incubated at 4°C overnight with primary antibodies, including anti-Gal-3 (M3/38; Santa Cruz), anti-Gal-8 (D-18; Santa Cruz), anti-parkin (ab15954; Abcam, Inc.), and anti-β-actin (AC-74; Sigma-Aldrich) antibodies. The membranes were then washed with 0.05% PBS–Tween 20 and incubated with a 1:5,000 dilution of horseradish peroxidase (HRP)-conjugated secondary antibody at room temperature for 1 h. After washing was performed, the membranes were soaked in ECL solution (PerkinElmer Life and Analytical Sciences, Inc.) for 1 min and exposed to an X-ray film (BioMax; Eastman Kodak). The relative levels of signal intensity were quantified using ImageJ software (version 1.41o; W. Rasband, National Institutes of Health). One set of representative data obtained from three independent experiments is shown, and the data are shown as means ± standard deviations (SD) of the results of three independent experiments.

### Plasmid overexpression.

Transient transfection was performed using TurboFect cell transfection reagent (Thermos) according to the manufacturer’s instructions for optimization and usage. The plasmids expressing HA-tagged pcDNA3-Gal-3 and its control, HA-tagged pcDNA3, were generated as described previously (70). pEGFP-Gal-3 and its control, pEGFP-N1, were obtained from F. T. Liu (Glycocore, IBMS, Academia Sinica) (71).

### RNA interference.

Selective gene expression was downregulated using lentiviral expression of short hairpin RNA (shRNA) targeting human Gal-3 (clone 1, TRCN0000029304 containing shRNA target sequence 5′-GCTCACTTGTTGCAGTACAAT-3′; clone 2, TRCN0000029306 containing shRNA target sequence 5′-GCAAACAGAATTGCTTTAGAT-3′; clone 3, TRCN0000029307 containing shRNA target sequence 5′-GCAGTACAATCATCGGGTTAA-3′), human Gal-8 (clone 1, TRCN0000057354 containing shRNA target sequence 5′-CCTGGAACTTTGATTGTGATA-3′; clone 2, TRCN0000057355 containing shRNA target sequence 5′-GCAAAGTGAATATTCACTCAA-3′; clone 3, TRCN0000419140 containing shRNA target sequence 5′-GGGTCCTCTGGGATTAGTTAT-3′), human parkin (clone 1, TRCN0000355824 containing shRNA target sequence 5′-GGCCTACAGAGTCGATGAAAG-3′; clone 2, TRCN0000355822 containing shRNA target sequence 5′-TTGCACCTGATCGCAACAAAT-3′; clone 3, TRCN0000355823 containing shRNA target sequence 5′-CGTGATTTGCTTAGACTGTTT-3′), and a negative-control construct (luciferase shRNA [shLuc]). The shRNA clones were obtained from the National RNAi Core Facility, Institute of Molecular Biology/Genomic Research Center, Academia Sinica, Taipei, Taiwan. In brief, HMEC-1 cells and A549 cells were transduced by lentivirus, with an appropriate multiplicity of infection (MOI), in complete growth medium supplemented with Polybrene (Sigma-Aldrich). After transduction for 24 h and puromycin (Calbiochem) selection for 6 days, protein expression was monitored using Western blot analysis.

### Immunoprecipitation.

Cells were lysed in lysis buffer and then centrifuged at 13,000 rpm for 20 min at 4°C. The protein concentration of the lysate was determined by Bradford assay. Antibody-coated protein G agarose beads were incubated with 300 μg lysate from each sample for 16 h at 4°C. The bead-protein complex was pulled down by centrifugation at 13,000 rpm and then washed five times with PBS-T (0.01% Tween 20–PBS). Samples were boiled for 10 min at 95°C and subjected to SDS-PAGE followed by Western blotting and protein detection.

### Mice and air pouch infection model.

Female C57BL/6JNarl wild-type (WT) and Gal-3-knockout (Gal-3^−/−^) mice (10 weeks of age), generated as described previously (72), were obtained from the National Laboratory Animal Center in Taiwan and the La Jolla Institute for Allergy and Immunology (LIAI), respectively. The LIAI authorized the Glycocore, IBMS, Academia Sinica, to transfer the material to others. The mice were maintained and manipulated according to the animal experiment guidelines of the Ministry of Science and Technology, Taiwan. The air pouch model of infection was established as previously described (73, 74). Briefly, mice were subcutaneously injected with 2 ml of air to form an air pouch and were inoculated with 2 × 10^8^ CFU of M49 strain NZ131. The local tissue damage area was photographed and measured by ImageJ software. At 48 h postinfection, air pouch exudates were collected by injecting 1 ml PBS into the air pouch and aspirating the exudates. The liver and spleen were further homogenized in 1 ml PBS. Bacterial colonies were quantified by counting on TSBY plates.

### Florescence immunohistochemistry staining.

The skin tissue with lesion was embedded with Tissue Tek OCT compound (Sakura), snap-frozen in liquid nitrogen, and stored at −80°C until analysis by immunohistology. The sections were then fixed with 4% paraformaldehyde; incubated with primary antibodies, including anti-GAS-fluorescein isothiocyanate (FITC) (ab68879; Abcam, Inc.) and anti-CD31 (550274; BD Biosciences) antibodies, at 4°C overnight; washed; incubated for 1 h with labeled isotype-specific secondary antibodies; washed; and counterstained with DAPI. The cells were visualized by the use of a confocal microscope (FV1000; Olympus).

### Statistical analysis.

Data obtained from three independent experiments were presented as means ± SD. Statistical analysis was performed using Prism version 5 (GraphPad Software, Inc.). Analysis of pairs of sets of data was performed using an unpaired Student’s *t* test. Analysis of three or more sets of data was performed using one-way analysis of variance (ANOVA) with Tukey’s multiple-comparison posttest. Statistical significance was set at a *P* value of <0.05.
