# Supplementary figures and images for: Galectin-3 Inhibits Galectin-8/Parkin-Mediated Ubiquitination of Group A Streptococcus
Source: mBio. 2017 Jul 25;8(4):e00899-17. doi: 10.1128/mBio.00899-17 (PMC5527311; doi:10.1128/mBio.00899-17)

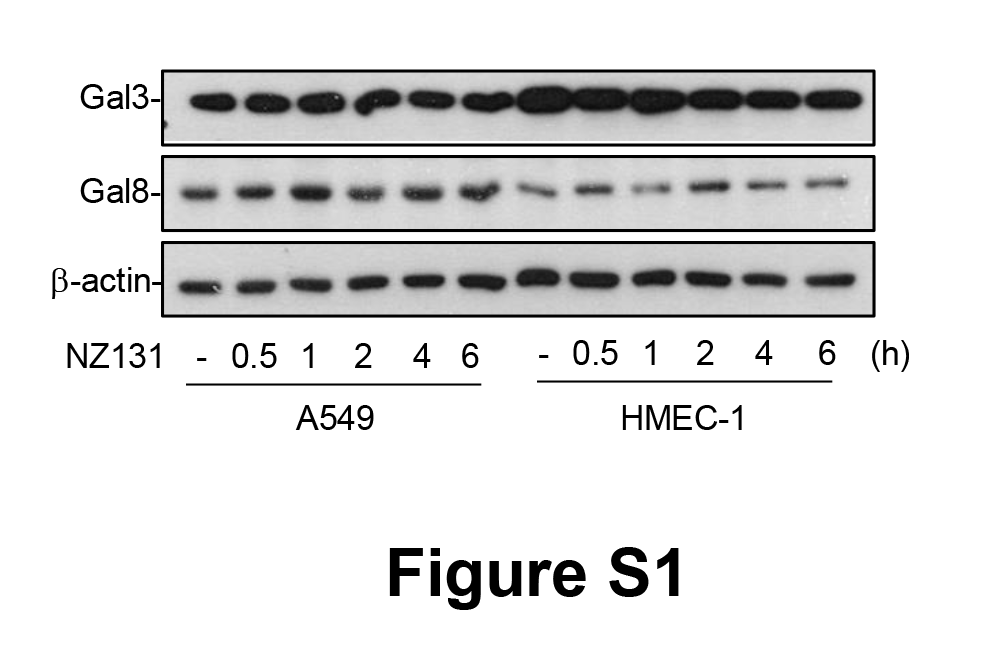

Supplement: FIG S1 [file mbo004173402sf1.tif]

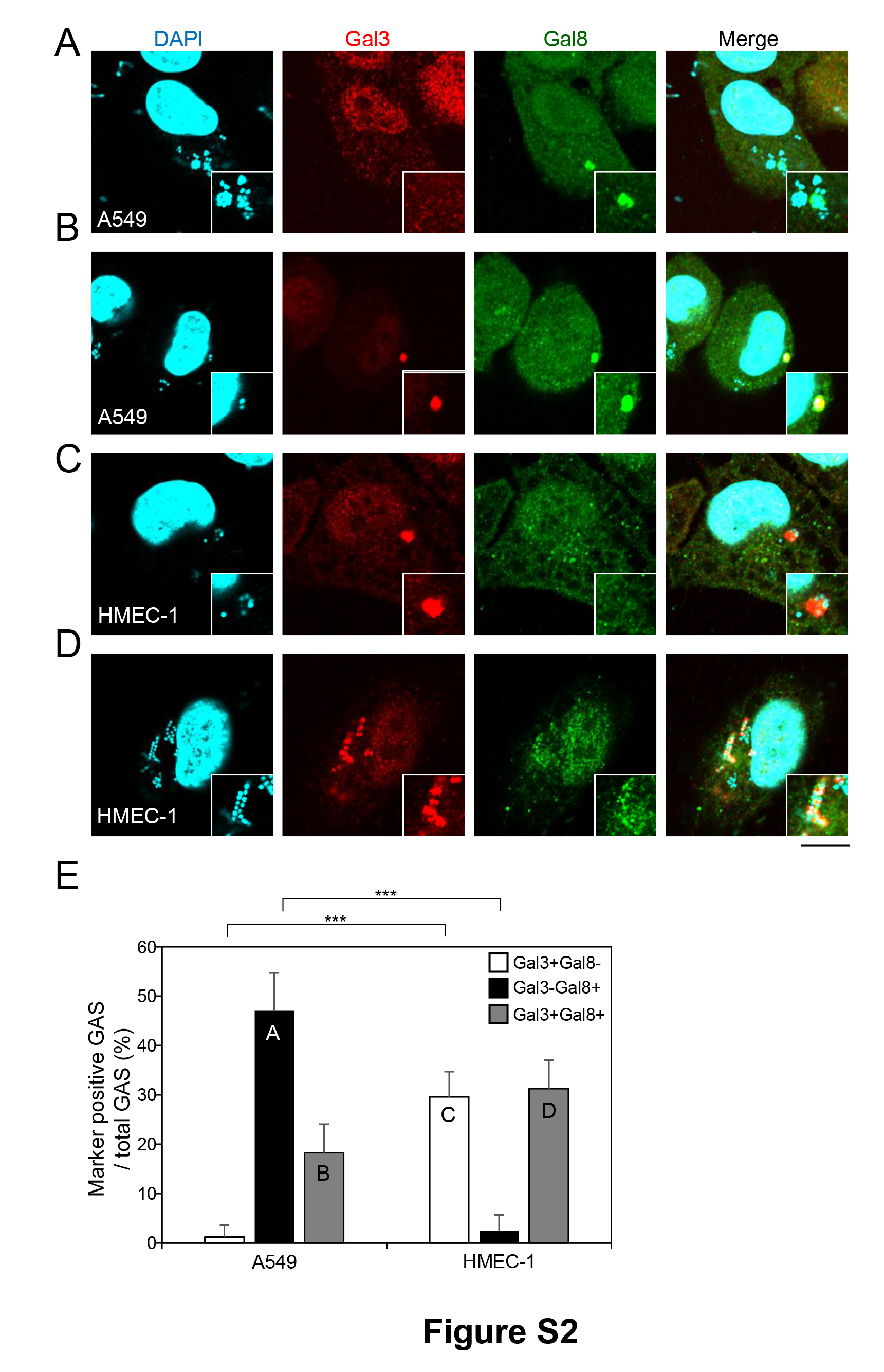

Supplement: FIG S2 [file mbo004173402sf2.tif]

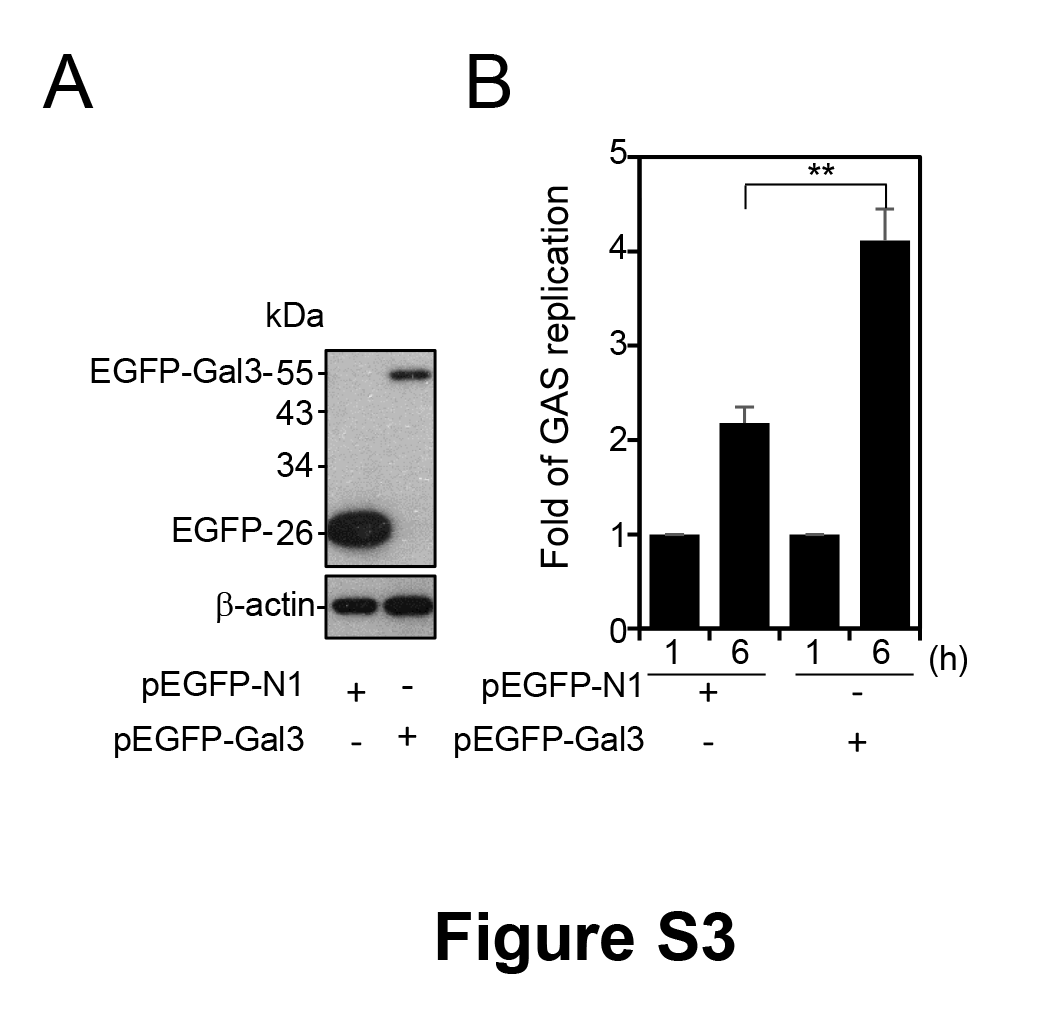

Supplement: FIG S3 [file mbo004173402sf3.tif]

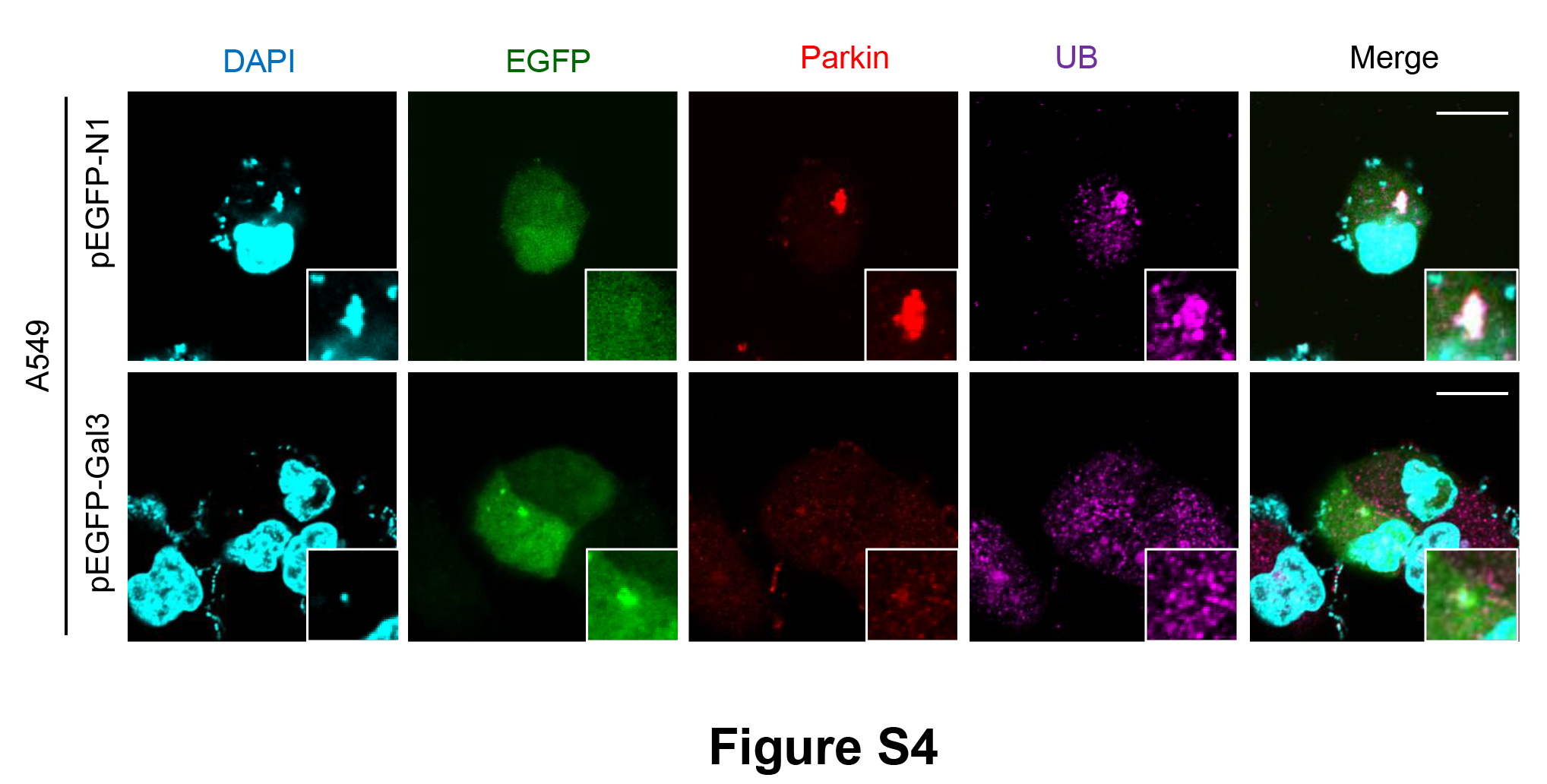

Supplement: FIG S4 [file mbo004173402sf4.tif]

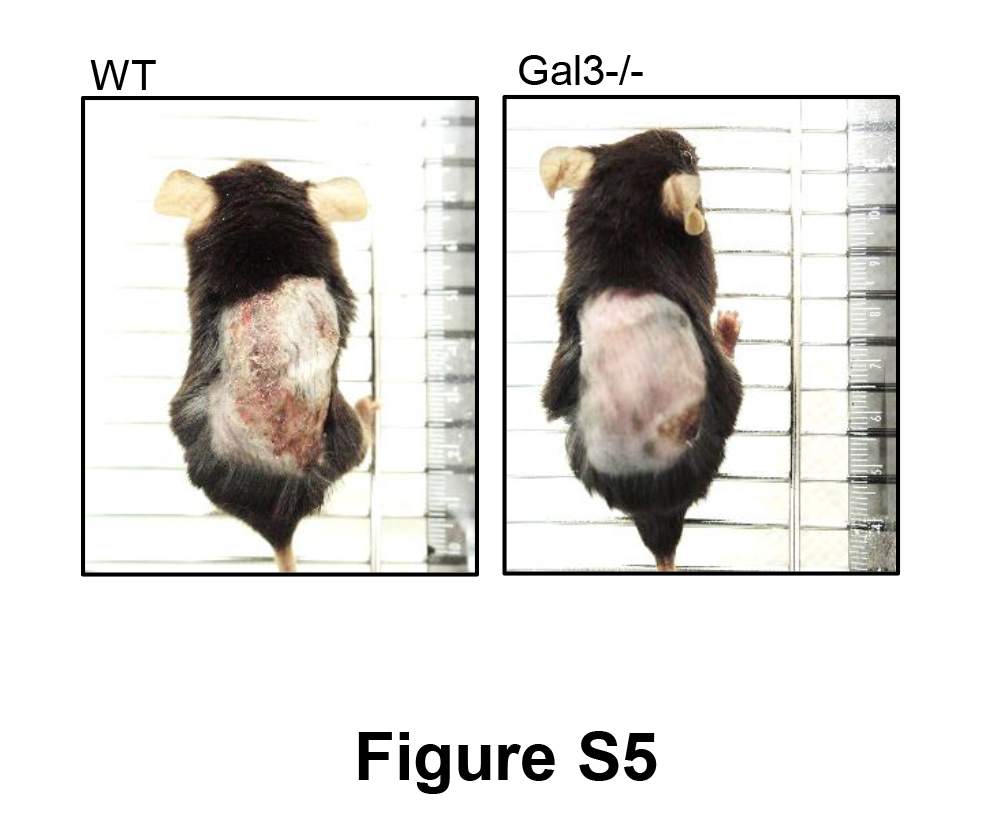

Supplement: FIG S5 [file mbo004173402sf5.tif]
